# Classifying health-related quality of life outcomes of total hip arthroplasty

**DOI:** 10.1186/1471-2474-6-48

**Published:** 2005-09-06

**Authors:** Min Xu, Donald S Garbuz, Lisa Kuramoto, Boris Sobolev

**Affiliations:** 1 Arthritis Research Centre of Canada, Vancouver, BC, Canada; 2 Department of Orthopaedics, University of British Columbia, Vancouver, BC, Canada; 3 Centre for Clinical Epidemiology & Evaluation, Vancouver, BC, Canada

## Abstract

**Background:**

Primary total hip arthroplasty (THA) is an effective treatment for hip osteoarthritis, assessed by whatever distribution-based measures of responsiveness. Yet, the group level evaluation has provided very little evidence contributes to our understanding of the large variation of treatment outcome. The objective is to develop criteria that classify individual treatment health related quality of life (HRQOL) outcome after primary THA, adjusted by preoperative scores.

**Methods:**

We prospectively measured 147 patients' disease specific HRQOL on the date of consultation and 12 months post operation by Western Ontario McMaster Universities Osteoarthritis Index (WOMAC). Regression models were used to determine the "expected" outcome for a certain individual baseline score. The ceiling effect of WOMAC measurement is addressed by implementing a left-censoring method.

**Results:**

The classification criteria are chosen to be the lower boundary of the 95% confidence interval (CI) of the estimated median from the regression. The robustness of the classification criteria was demonstrated using the Monte-Carlo simulation.

**Conclusion:**

The classification criteria are robust and can be applied in general orthopaedic research when the sample size is reasonable large (over 500).

## Background

Statistical tests are frequently called upon to assess treatments whose effect size is small or whose reduction of risk is modest, as is often the case with emerging treatments. But what of the evaluation of mature treatments whose effect is known to be substantial?

Primary total hip arthroplasty (THA) is an effective treatment for patients with severe hip osteoarthritis (OA). The improvement is large by any of the measures of responsiveness commonly used in orthopaedic research [1-6]. Yet, there are large variations reported in treatment outcome and very little good-quality evidence contributes to our understanding of this variation [7,8]. The evidence is limited mostly to patient- and implant-related factors [7]. The role of variations in service delivery practices and other factors remains unclear. On the whole, patients' outcome is good. Nevertheless, the development of classification criteria to differentiate between overall good results is necessary to achieve a better understanding of the variance and ultimately to reduce the number of relatively poor performers.

Because THA aims to improve physical function and relieve pain, and because it is broadly successful in its goal, health-related quality of life (HRQOL) is generally acknowledged to be the primary outcome of interest [9,10]. Assessment of HRQOL is typically made at the group level, that is, by measures such as the t-test, effect size (ES), and standard response mean (SRM), that are characteristic of a group. However, using such methods may not provide the best evidence to explain the association between postoperative outcomes and risk factors [8]. In contrast, the measurement of individual changes is an increasingly attractive method of quantifying HRQOL outcomes because it has the potential to document objectively the patient-perceived impact of treatment. Expectation and satisfaction are highly individualized; they contribute significantly to self-assessed quality of life. But these individualizing influences are lost in statistics such as pre- and postoperative mean scores that only express a group [11]. Raising the mean outcome is a worthwhile objective, especially when the mean badly needs improvement. When, as with effective treatments, the mean is not an overriding concern, it is appropriate to turn our attention to individuals within the mean [12]. Even groups whose mean change due to treatment is equivalent are likely to contain individuals who did substantially better and worse than others [13]. Developing statistical methods to assess these differences rather than the means themselves is a natural accompaniment to the refinement of treatments such as hip arthroplasty.

Two recent studies have found an association between preoperative health status and postoperative outcomes [14-16]. Fortin et al. examined the relationship between preoperative functional status and postoperative outcomes in a prospective cohort study using the Western Ontario McMaster Universities Osteoarthritis Index (WOMAC) and Short Form 36 (SF-36). They found that poorer preoperative function was the strongest predictor of pain and functional outcomes at 6 and 24 months after THA [15,16]. The authors concluded that surgery performed later in the natural history of functional decline results in worse postoperative functional status. They also noted that function and pain in patients with lower preoperative function did not improve after the operation to the level achieved by those with higher preoperative scores [15,16]. Thus, measures of postoperative HRQOL outcome need to be adjusted by preoperative functional status.

## Methods

The objective of this study is to develop a tool for classifying the HRQOL outcome of THA based on the individual's preoperative HRQOL score. The development of the tool and the results of a simulation study are presented in the methods section. We describe the design of a case study evaluating the postoperative outcome for THA. In the development of the instrument section, a left-censored linear regression model is employed as a means of understanding and communicating the relationship between baseline and expected outcome. An expected postoperative HRQOL score for each individual preoperative score is estimated using this left-censored linear regression model. By using the expected HRQOL outcome, we identify patients whose benefit from THA is "better than expected." The performance of these classification criteria is evaluated in difference sample sizes by simulation. In the development of these classification criteria we adjust the postoperative outcome by its preoperative score. The result of this simulation study shows that these classification criteria are robust.

### Study population

Data from a prospective cohort study were used for a case study. This study included 201 patients registered on the wait list for THA between March, 2001 and May, 2003 with 147 patients completed follow-up ending in March, 2004. This study was conducted at the Vancouver Hospital & Health Sciences Centre. Ethical approval was issued by the University of British Columbia Clinical Ethics Review Board. Patients presenting during this period at the Division of Reconstructive Orthopedics at Vancouver Hospital (VH) with a diagnosis of osteoarthritis (OA) and requiring primary THA are included in the study. OA is defined by the American College of Rheumatology's (ACR) clinical classification criteria for OA of the hip [17]. Patients were excluded for the following reasons: previous THA to the index joint; inflammatory arthritis; bilateral THA performed simultaneously; inability to respond to a questionnaire in English; and urgent surgery performed within 28 days after the decision for THA.

Every patient requiring hip arthroplasty was requested to complete the WOMAC questionnaire on the date of consultation. The questionnaire is self-administered. Medical office assistants handed each patient a WOMAC questionnaire once the decision was reached to enter the wait list. To assess postoperative outcomes, WOMAC questionnaires were mailed at 12 months following surgery. WOMAC is recommended for OA-specific outcomes [18,19]. It contains dimensions for pain (5 items), stiffness (2 items), and function (17 items). Dimensions are equally weighted and reported as sums, where the higher number indicates a greater burden of OA. At present it is the most frequently used measure of pain and self-reported disability among arthroplasty patients [10]. The WOMAC questionnaire has 24 questions, each question is given a Likert scale response from 0 (best health state) to 4 (worst health state). The WOMAC score for each subscale is calculated as the sum of the scores of each question included in the subscale. The range of each subscale is as follows: function: 0–68; pain: 0–20; stiffness: 0–8.

Patients' names and provincial health numbers were used to obtain age and gender through the medical office administrative database. Co-morbidity information was obtained through medical chart review using the Charnley classification, which stratifies patients by the presence of OA in one or both hips, or a co-morbid condition that impairs walking. This scale allows a meaningful comparison between groups [20]. The Charnley classes we used are:

A: Single hip with osteoarthritis

B1: Bilateral hips with arthritis

B2: Previous THA on the contra-lateral hip

C: Multiple joints affected with arthritis or a chronic disease that affects HRQOL (specifically walking)

### Statistical analysis

#### Log-linear regression model

**Figure 1:**
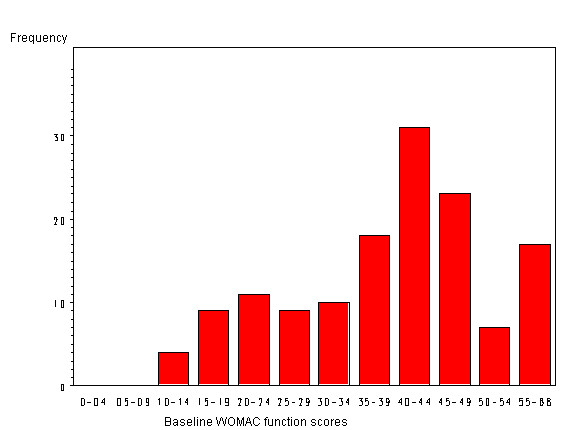
The distribution of baseline WOMAC functional scores.

**Figure 2:**
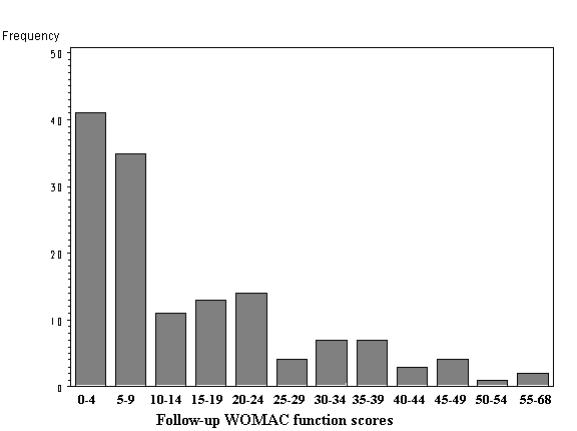
The distribution of follow-up WOMAC function scores.

**Figure 3:**
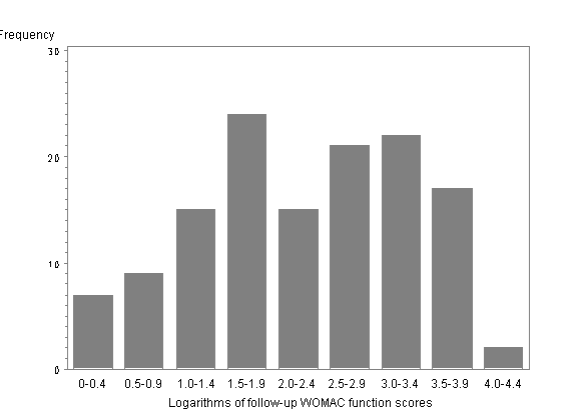
The distribution of the logarithms of follow-up WOMAC functional scores.

In the following, we aim at building a linear model to explore the relationship between follow-up score and baseline score. Since the distribution is skewed (Fig. 1 & Fig. 2), one cannot use the follow-up score in a linear regression analysis as an outcome variable. We found that the logarithms of the follow-up WOMAC functional scores follow a symmetrical distribution (Fig. 3). Therefore we build a log-linear regression as following:

log(Follow-up) = α+β*Baseline+σ*ε,

where *Follow-up *is the follow-up WOMAC score and *Baseline *is the baseline WOMAC score. The error term ε follows a normal distribution with a mean of 0 and a standard deviation of 1, and σ is a fixed constant that changes the variability of the expected value.

However the observed follow-up data has some WOMAC function score equal to zero and the logarithm of 0 is infinite. So we can not censor the postoperative score at 0. Moreover, the WOMAC function 0 is corresponding to complete freedom from joint symptoms. It is unlikely that patients before and after THA would have no detectable impairment in their hip. Therefore, as a measurement tool, the WOMAC questionnaire is limited in providing HRQOL information at the extreme low end of the scale (score of 0). Since a true score is unknown when the score is between 0 and 1, we regard measurements below 1 as left-censored observations. In our model, we chose 0.9 to be the censoring point so that 1 was preserved in the model and 0 was censored. We transformed the observed follow-up score as follows:

*Follow-up *= *0.9*, *if Follow-up *<= 0.9;

*Follow-up *= *Follow-up*, *if Follow-up > 0.9*,

The Tobit regression model is a well known instrument for measuring left-censored variables in economic research [21]. In order to incorporate the left-censored observations in the regression analysis, we built a Tobit model to incorporate the left-censored observations in the regression analysis. The maximum likelihood method was used to estimate the probabilities of log(*Follow-up*) given the baseline WOMAC score. The regression analysis was conducted using the SAS 8.1 PROC LIFEREG procedure.

#### Instrument for classifying function outcomes

Through this regression analysis, an expected postoperative outcome for each baseline WOMAC functional score was obtained. Due to the skewed distribution of follow-up scores, we used the median of the follow-up score instead of the mean as the classification criteria. The mean of the predicted logarithm of follow-up scores was estimated through the model, and the median of estimated follow-up scores is exp (mean of log(*Follow-up*)) according to the mathematical transformation.

Since the model is derived from a rather small size sample, the variation of the estimated median of the follow-up scores should be taken into consideration. Using the lower 95% confidence interval of the median as a cutoff point associated with the baseline score, the study patients were divided into two groups. Group I: *Patients below the line were considered to have achieved a "better than expected" outcome*. Group II: *Patients above the line were considered to have achieved a "not better than expected" outcome*.

#### Assessment of the classification instrument

We implemented the Monte-Carlo simulation method to investigate the robustness of our classification criteria. Our intention was to assess the robustness across baseline scores and for different sample sizes. We generated random postoperative WOMAC functional scores assuming a systematic relationship between the baseline scores and postoperative WOMAC functional scores and adding a random component. The systematic relationship and the parameters for the random component were specified from the Tobit model estimates in our case study. In each data set, postoperative scores were generated for baseline WOMAC functional scores fixed at 10, 17, 34, 51, and 68. We chose 10 since it is the lowest baseline score that is eligible for surgery and available in the case study. The functional subscale contains 17 questions; each question has a response on Likert scale from 0 to 4. Therefore, we chose the folds of 17 as the baseline levels for simulation. The postoperative score was left-censored at 0.9. We looked at sample sizes increasing from 100 to 500 in increments of 100. For each sample size, we generated 1000 data sets. Then, for each data set, the regression model was fit and the median postoperative score and the cutoff points (ie. the lower bound of the 95% CI for the median score) were estimated at each baseline score.

We also tested the model using same method with different censoring points (0.9, 2, 3, 4, and 5) while the sample size was fixed at 500. For each censoring point, we generated 1000 datasets and the cutoff points were estimated for each data set.

## Results

### Study population

This study included 201 patients, among which there are 147 patients completed follow-up ending in March, 2004. The average age is 64.8 years and there are 83 females (56%) and 66 males (44%) in the study. Seventy-two patients (50%) have only one joint involved with OA; 34 patients have bilateral disease. Of these 34 patients, there are 18 with contra-lateral hip replacement prior to the index surgery and 16 patients with moderate to severe OA in the contra-lateral hip. Thirty-nine patients (27%) have multiple joints involved with OA or have a chronic systematic disease. When compare the component of age, gender and disease statues, there are no statistical differences of between the 147 patients and 54 patients who did not complete follow-up. In the following analysis, all the results are based on the 147 patients who completed follow-up. We found that the distribution of baseline WOMAC functional scores (scale 0–68) follows a symmetrical distribution and its' mean and standard deviation (SD) are 39 and 13 respectively (Fig. 1). Its minimum is 10 points and median is 41 points. While at the end of the follow-up, the distribution of WOMAC functional scores (scale 0–68) shows a truncated distribution because the follow-up outcome is nearly as good as a full recovery or normal function; that is, the follow-up outcomes have a limit as a score of 0 (best function). The mean follow-up WOMAC functional score is 14 (SD = 14). Its minimum is 0 points and median is 8.5 points. Since the distribution is skewed, one cannot use the follow-up score in a linear regression analysis as an outcome variable.

#### Log-linear regression model

Table 1 shows the parameter estimates obtained through this regression analysis. For the sample population, the estimate of the expected value of the lognormal distribution is given by:

**Table 1:** Parameter estimation for the log-linear regression

**Parameters**	**Estimates**	**95% CI**
**Intercept**	0.98	0.29–1.67
**Coefficient**	0.03	0.01–0.05
**Scale**	1.30	1.14–1.48

Log(Follow-up) = 0.98+0.03*Baseline.

**Figure 4:**
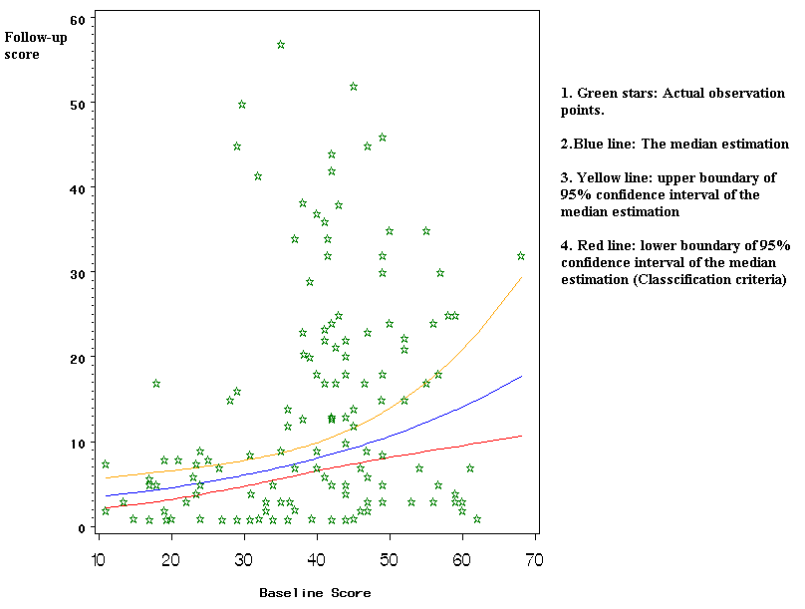
95% confidence interval for the median of expected function outcomes.

Based on the model, the baseline WOMAC functional score is a significant predictor of the follow-up WOMAC functional score (p = 0.0005). Increasing the baseline score by 10 points raises the estimated postoperative score by approximately 35%. The estimated median score line and its 95% confidence interval are shown in Fig. 4.

Age, gender, co-morbidity, and waiting time were also tested as covariates in the log-linear regression model. None of these variables were significant predictors of the follow-up WOMAC functional score and there was no difference in the regression coefficient for baseline WOMAC functional scores with or without these covariates. A goodness of fit test showed that the Tobit model is well fitted. In the model, the outliers are detected by the studentized residual; those observation having an absolute studentized residual over 3.5 were removed.

#### Instrument for classifying function outcomes

**Figure 5:**
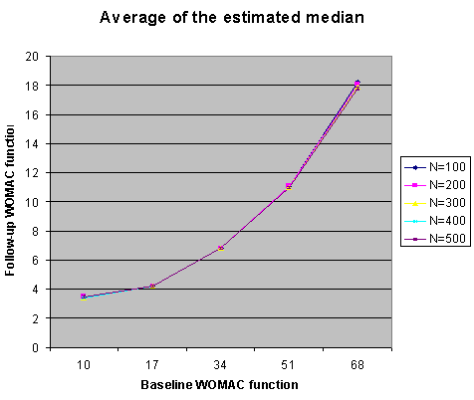
The average of the median estimates from simulation.

**Figure 6:**
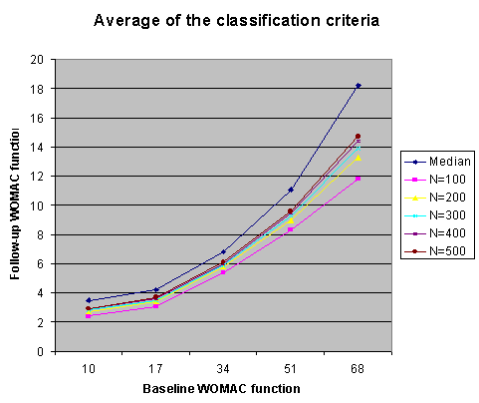
Average of the estimated median and classification criteria.

**Figure 7:**
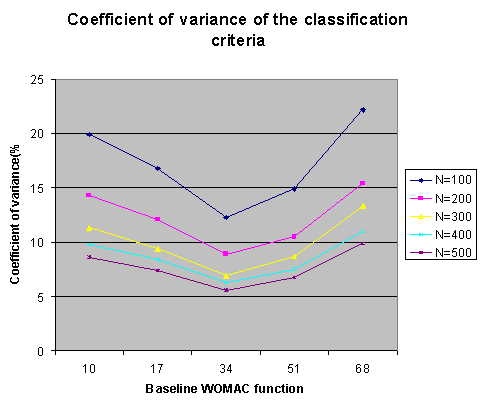
The coefficient of variation of the classification criteria from simulation (Different sample size).

**Figure 8:**
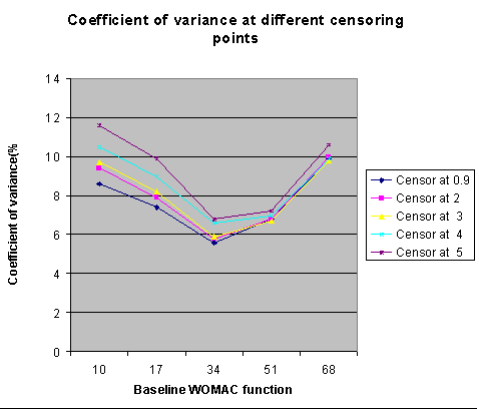
The coefficient of variation of the classification criteria from simulation (Different censor point).

The simulation results are summarized in Fig. 5, 6, 7, 8 and Table 2. Fig. 5 summarizes that the median estimation is very consistent despite the increase in sample size. Table 2 represents the same information as Fig. 5, but provides the actual values for classification criteria that can be used as a reference table for future researchers.

**Table 2:** Average of the classification criteria

	**Average of the classification criteria**				
**Baseline**	**N = 100**	**N = 200**	**N = 300**	**N = 400**	**N = 500**

10	3.5	3.5	3.4	3.4	3.5
17	4.2	4.2	4.2	4.2	4.2
34	6.8	6.8	6.8	6.8	6.8
51	11.1	11.1	11	11	11
68	18.2	18.1	17.9	17.8	17.8

Fig. 6 summarizes the results of classification criteria in simulated data sets with different sample size. While the sample size increases, the mean of the cutoff points approaches the mean of the median estimation. That is, when the sample size is reasonable large (n = 500), the cut off points are almost equivalent to the estimated median.

Fig. 7 summarizes the coefficient of variation (CV) for the distribution of classification criteria, in simulated data sets with different sample size. The lower the CV is, the higher the precision of the estimation is. This plot shows two trends. First, the CV is lowest at the median baseline level and increases toward the extreme values in both directions, as expected. For example, in a sample of 100 patients, the CV of the estimated cutoff point is 12.5% at baseline 34, 20.2% at baseline 10 and 22.8% at baseline 68. That is, the precision of this estimation is the highest when the baseline is around 34 and reduced toward both extremes. Second, we also found that the CV decreases with the sample size. For example, at baseline 34, the CV is 12.5% for 100 scores and 5.3% for 500 scores. This indicates that the precision of the estimation increases with a larger sample size.

Fig. 8 summarizes the CV for the distribution of classification criteria, in simulated data sets with different censoring points. While sample sizes being fixed at 500, we found that the CV increases with higher censoring level. This indicates that the precision of the estimation increases with a lower censoring point. Therefore censoring postoperative scores at 0.9 is preferred over censoring at a higher level.

## Discussion

In the past decade the orthopaedic community has shifted toward the inclusion of patient-based measures of outcome assessments [22]. It was typical of earlier orthopedic practice that the patient's perspective received less attention than did clinician's measures of disease and impairment [23,24]. Clinicians used complication rates, mortality, most frequently revision rates and clinical judgment to assess the degree of improvement [25]. Since THA, in most cases, aims explicitly to improve HRQOL, using HRQOL measures as endpoint in orthopaedic research on evaluation of treatment outcome is now seen as a necessity to fully understand the effects of this intervention [26].

THA is an effective treatment by any of the distribution-based measures of responsiveness [6]. Yet, there are large variations reported in treatment outcome. Why some patients do better than others post-operation? Group level perspective on evaluation of treatment outcome has provided very little evidence contributes to our understanding of this question.

Most reports of the HRQOL outcome of THA use distribution-based approaches that test the significance of the change due to treatment [22]. However, distribution-based approaches are based on the statistical characteristics of the sample. For example, paired t-statistics are frequently used to estimate the statistical significance of the change [27]. The problem with using the t-test as a measure of change is that it focuses exclusively on the significance which will inevitably increase with sample size [28]. A different problem exists in determining the minimal clinically significant difference for THA. In clinical drug trials, a 9.3 point change in WOMAC functional score was accepted as a minimally significant improvement in arthritis symptoms [29]. But the 9.3-point change is too small to be applied to the outcomes of THA which typically show a 60–100% improvement over baseline [15,16]. The expected change in WOMAC functional scores after THA is four times larger than the minimal clinically important difference derived from drug trials in OA. Effect size (ES) and standard response mean (SRM) are also common measures for responsiveness at the group level. Cohen's criteria can be used to classify responsiveness as mild, moderate, and large [30]. But these statistics may be influenced by the heterogeneity of the sample. Moreover, Cohen's magnitude of effect does not suit the nature of orthopaedic surgery. An effect size larger than 0.8 is considered a "large effect". However, by that criterion, the majority of patients in our case study would be considered to have experienced a "large effect" both by ES and SRM statistics. Such criteria are inadequate for documenting the positive impacts of treatment. Characteristics of the baseline distribution will strongly influence the effect size, while variability of the change in the sample may influence the standard response mean.

We developed a method to classify the HRQOL outcome on an individual level. Group distributions can have a negligible mean difference with large variance. Therefore, the large differences that are important to individuals are not measured by group level, whereas the individual level takes them into account. This makes the individual perspective important for clinical treatment decisions [13,28]. We have shown that improvement after hip arthroplasty is not as big when the patients have a better preoperative score; therefore, postoperative outcomes are not evaluated at the group level but rather at each individual baseline level, so that for each individual patient an expected outcome can be generated.

We addressed the ceiling effect of the WOMAC instrument in the measurement of postoperative outcome of THA, as 10% of patients in our case study recorded a postoperative WOMAC score of 0. A ceiling effect occurs when a patient can improve only minimally or not at all. In the presence of a ceiling effect, the paper by Austin et al. suggests that the coefficient estimates from the left-censored regression model are better than the estimates from a least square regression [31]. We address the ceiling effect by implementing a linear regression of log-transformed WOMAC function score while treating postoperative scores as left-censored at 0.9. The regression model represents the relationship between baseline and postoperative outcome.

The estimated median of postoperative scores was chosen to distinguish between those who are able to benefit fully from treatment and those who are not. Due to the small sample size, the classification criteria in this case study is the lower boundary of the 95% confidence interval (CI) of the estimated median. Our study results agrees with the previous literature in that postoperative HRQOL scores were found to be strongly associated with their baseline values. We evaluated the changes in the WOMAC dimensions of pain, stiffness, and function from pre- to post operation. The effects of age, gender, and co-morbidity on follow-up WOMAC scores were not statistically significant, so these are excluded from the regression model.

The performance of the classification criteria was demonstrated using the Monte-Carlo simulation. The variation of the classification criteria will decrease with increasing sample size; likewise, the classification criteria become closer to the estimated median with increasing sample size. Thus, with a small sample set, researchers could use the lower boundary of 95% CI of the estimated median as the classification criteria. When there is a reasonable larger sample (bigger than 500), one could use the estimated median itself as the classification criteria.

The limitation of this research is that the estimated classification criteria were not validated in a different clinical setting. Instead, they were evaluated through simulation. Therefore, we are recommending that clinicians use only the methods rather than the actual values of the classification criteria until further research is done in this area.

## Conclusion

The contribution of this paper is two-fold. First, the criteria for classify individual treatment outcome adjusted by baseline score was proposed. The development of these classification criteria also addresses the ceiling effect of the HRQOL measurement. Second, the performance of the classification criteria was found to be more precise with a reasonable larger sample size (n > 500). Vancouver Hospital (VH) is a tertiary referral centre and teaching hospital for the University of British Columbia (UBC). The demographics of arthroplasty patients, however, are not different from elsewhere. The study result is expected to be generalizable to a similar clinical setting.

This paper provides intuitive criteria for classifying HRQOL outcomes based on individual scores before surgery. The result of this method is an individual outcome which can serve as a standard advice for patient counseling based on HRQOL status at consultation. It gives orthopaedic researchers a means of defining "success" of effective surgery. In the future, we will evaluate this method in different populations and with other HRQOL instruments such as Oxford Hip Score and the Short Form 12 questionnaire.

## Competing interests

The author(s) declare that they have no competing interests.

## Authors' contributions

Analysis of data, interpretation and the original draft were completed by Min Xu. Donald Garbuz conceived the study, participated in the design and contributed to clinical conception and interpretation. Lisa Kuramoto performed the Monte-Carlo simulation in the statistical analysis. Boris Sobolev also participated in the design, provided critical evaluation of methodological content and revision of the manuscript. All authors read and approved the final manuscript.

## Pre-publication history

The pre-publication history for this paper can be accessed here:


